
JOURNAL INFORMATION
==============================
NLM Title Abbreviation: Innov Aging
Journal ID: Innov Aging
NLM Journal ID: 101703706
Journal ID: innovateage

Innovation in Aging

EISSN: 2399-5300
Publisher: Oxford University Press

ARTICLE INFORMATION
==============================
PMCID: PMC12759766
DOI: 10.1093/geroni/igaf122.678
Article ID: igaf122.678
Article version: 1
Subjects: Session 2137 (Biological Sciences Invited Symposium), AcademicSubjects/SOC02600

Presidential Symposium: Integrating Geroscience Across Scale

Curran Sean Chair
Publication date: 2025 Dec
Electronic publication date: 2025 Dec 31
Volume: 9
Issue: Suppl 2
Issue title: Program Abstracts from the GSA 2025 Annual Scientific Meeting, “Innovative Horizons in Gerontology”
Electronic Location ID: igaf122.678
Copyright: © The Author(s) 2025. Published by Oxford University Press on behalf of the Gerontological Society of America.
Copyright year: 2025
License: This is an Open Access article distributed under the terms of the Creative Commons Attribution License (https://creativecommons.org/licenses/by/4.0/), which permits unrestricted reuse, distribution, and reproduction in any medium, provided the original work is properly cited.
License URL: https://creativecommons.org/licenses/by/4.0/

Page count: 1

==============================

### SESSION 2137 (BIOLOGICAL SCIENCES INVITED SYMPOSIUM)

Abstract citation ID: igaf122.678

**PRESIDENTIAL SYMPOSIUM: INTEGRATING GEROSCIENCE ACROSS SCALE**

Chair: Sean Curran
